# Adolescent Male Couples-Based HIV Testing Intervention (We Test): Protocol for a Type 1, Hybrid Implementation-Effectiveness Trial

**DOI:** 10.2196/11186

**Published:** 2019-06-07

**Authors:** Tyrel J Starks, Sarah W Feldstein Ewing, Travis Lovejoy, Sitaji Gurung, Demetria Cain, Carolyn A Fan, Sylvie Naar, Jeffrey T Parsons

**Affiliations:** 1 Center for HIV Educational Studies and Training Hunter College City University of New York New York, NY United States; 2 Hunter College Department of Psychology Hunter College City University of New York New York, NY United States; 3 Health Psychology and Clinical Science Doctoral Program Graduate Center City University of New York New York, NY United States; 4 Department of Psychiatry Oregon Health & Science University Portland, OR United States; 5 Center to Improve Veteran Involvement in Care VA Portland Health Care System Portland, OR United States; 6 College of Medicine Florida State University Tallahassee, FL United States

**Keywords:** men who have sex with men, adolescents, HIV, comparative effectiveness research

## Abstract

**Background:**

Young men who have sex with men (YMSM), particularly those who are partnered, are at unique risk for HIV. YMSM are among those at highest risk for HIV. Meanwhile, despite the fact that primary partners account for many—possibly most—new HIV infections, partnered men who have sex with men perceive themselves to be at much lower risk for HIV infection and therefore test less often than single men. In response to the risk of primary partner HIV transmission, couples HIV testing and counseling (CHTC) procedures have been developed for use in adult populations. Although promising, YMSM couples may require additional support to complete CHTC given their developmental context in which sexual and romantic relationships are relatively new, and communication skills are emergent.

**Objective:**

The aim of this study was to test the additive benefit of adjunct treatment components tailored for YMSM, which enhance communication skills before the completion of CHTC. The intervention tests a continuum of prevention packages including assertive communication training videos and motivational interviewing focused on assisting with identification and development (MI-AID) before entering into the dyadic intervention components. This protocol is part of the Adolescent Medicine Trials Network (ATN) Scale It Up program described in this issue.

**Methods:**

This is a comparative effectiveness trial that will be executed in 3 phases. Phase 1 will gather qualitative data related to intervention development and implementation from partnered YMSM at 4 subject recruitment venues (SRVs). Phase 2 will compare a continuum of these interventions in a pilot randomized controlled trial (RCT) at 2 SRVs. Phase 3 will compare the most successful adapted intervention package from phase 2 to CHTC as usual in a larger RCT at 4 SRVs. This phase is focused on implementation and sustainment phases of the Exploration, Preparation, Implementation, and Sustainment framework.

**Results:**

Phase 1 data will be drawn from qualitative interviews with partnered YMSM (n=24) and staff from ATN sites (n=20). Baseline enrollment for phase 2 is expected to begin across 2 SRVs in June 2018 (n_couples_=36). In phase 2, survey data collection along with HIV and sexually transmitted infection (STI) testing will occur at baseline, and 1- and 3-month (postintervention) follow-ups. Phase 3 will begin enrollment across 4 SRVs in September 2019 (n_couples_=144) and follow-ups will occur at 1, 3, 6, and 9 months postintervention.

**Conclusions:**

Although MI-AID, video-based assertive communication training, and CHTC have established efficacy when administered on their own, this study will be the first to evaluate the strongest adjunctive version of these interventions to address the specific developmental needs of partnered YMSM.

**Trial Registration:**

ClinicalTrials.gov NCT03386110; http://clinicaltrials.gov/ct2/show/NCT03386110 (Archived by WebCite at http://www.webcitation.org/75mlO7GCx)

**International Registered Report Identifier (IRRID):**

DERR1-10.2196/11186

## Introduction

### Background

Partnered young men who have sex with men (YMSM) are a uniquely vulnerable population. Adolescents in relationships continue to be an underexamined subgroup, despite the fact that YMSM are at the highest risk of HIV infection. Youth aged 13 to 24 years made up more than a fifth of new HIV diagnoses in 2015 [1]. The vast majority (81%) included gay and bisexual males [1]. Among men who have sex with men (MSM) aged 18 years and older, 35% to 68% of new HIV infections are transmitted between partners in primary (vs casual) relationships [2,3]. Primary partners account for 79% of new infections within the youngest MSM cohort included in Sullivan et al [3].

A number of factors may collectively contribute to the elevated risk of HIV transmission between primary partners. Gay men have anal sex—and receptive anal sex specifically—more frequently with primary (vs casual) partners [3]. Concomitantly, MSM are less likely to use condoms with primary partners. Condom use may be suppressed with primary partners because condomless anal sex (CAS) is interpreted as an indicator of commitment and emotional closeness [4-7]. A strong, positive association between CAS and relationship seriousness has been observed among YMSM [8]. Despite the incidence of main partner HIV transmission, partnered MSM perceive themselves to be at much lower risk of HIV infection and test for HIV less often [9,10]. In addition, for adolescent MSM, relationship development is still new, and romantic partnerships tend to be of short duration [8]. Thus, frequent brief primary partnerships, low perception of CAS risk, and the relative novelty of negotiating close relationships during this developmental period may escalate HIV infection among partnered YMSM.

Less than half of partnered adolescents feel comfortable advocating for and discussing condom use before sexual intercourse, contributing to 50% of youth consenting to condomless sex, despite wanting to use a condom [11,12]. YMSM show even lower rates of assertive communication relative to heterosexual age-mates [13]. Enhancing communication skills is thus a route to improve sexual safety in this age group [14,15].

### Theoretical and Evidence Base

Individual HIV prevention, including HIV testing, is conceptualized within the Self-Management Theory framework [16,17]. Problem solving, decision making, and access to care predict health behavior engagement. This, in turn, develops positive provider relationships that facilitate behavioral skills development and access to care.

Although useful, the framework does not fully address or incorporate the inherently interpersonal nature of sexual health decision making for individuals in relationships. The dyadic processes that contribute to the establishment and attainment of sexual health goals have been conceptualized within the framework of couples interdependence theory (CIT) [18-20]. Of the processes described within CIT, 2 are particularly useful when thinking about HIV risk reduction with couples [18,21]. Accommodation refers to a couple’s arrival at a shared or joint goal. Joint goals are more likely to be accomplished because they draw upon resources from both partners within the couple as they support each other in goal attainment. The transformation of motivation refers to the transition from considering primarily interpersonal gain and loss in decision making (and moments of conflict) to partners’ consideration of the consequences of their actions—not only for their own outcomes but for their partner and the overall health of their relationship.

Within CIT, each partner in the relationship can be viewed as having his own initial HIV-risk reduction and sexual health maintenances goals. CIT would suggest that couples who are able to accommodate potentially divergent health and safety preferences and arrive at a shared goal and strategy for its attainment have a greater potential to maximize their health outcomes [18,21-23]. A sexual agreement defines the rules and boundaries related to sex with partners outside the relationship [20,24]. They potentially encompass both the broad issue of whether casual partners are permitted and the rules that govern sex with casual partners when it occurs [25].

Couples HIV testing and counseling (CHTC) utilizes the concept of sexual agreement negotiations to catalyze the accommodation of partners’ sexual health goals. Over the course of a CHTC session, couples discuss their current HIV prevention practices, formulate a sexual agreement, and discuss how they might handle agreement violations. After receiving their HIV test result together, the couple formulates a shared HIV treatment or prevention plan in the context of their agreement [26,27].

Although a sound strategy, the negotiations inherent in the accommodation process assume that the individuals in the couple have adequate communication skills. To be effective, individuals must identify both their own and their partner’s preferences and feelings, and then communicate those concepts in constructive ways. To date, CHTC has been tested with adults [28]; this leaves the question of whether adolescents are similarly positioned to benefit from the intervention. As their communication skills are still very much in development [29], particularly in the relationship context [30,31], youth may need added support to effectively identify sexual goals and to learn to communicate them carefully and productively. Youth may need the support of additional communication and skills practice, both modeled (such as in assertive communication training, CT) or as shaped through actual role plays [32] to successfully convey their sexual and relationship goals to their likely, relatively new relationship partner.

Rosenthal and Starks [33] found that stigma directed at their relationship was associated with mental health functioning above and beyond stigma directed at gay men individually. Furthermore, their work showed that relationship functioning buffered against this negative association. Building on this work, there is evidence to suggest that providing models of YMSM communicating effectively with relationship partners may have beneficial effects on mood or anxiety. Studies of gay men in relationships suggest that being partnered may be associated with a range of mental health benefits [34-36]. A potential pathway for this links dyadic functioning and mental health. Dyadic research with gay couples has indicated that depression scores are predicted by both personal and the partner’s relationship satisfaction scores [37]. To the extent that improvements in communication skills result in improvements in dyadic functioning, it is plausible that mental health outcomes may also result in similar improvements. A second pathway, which might conceivably result in secondary effects on mood and/or anxiety, lies in the potential to reduce the effects of stigma by challenging negative stereotypes about gay men and gay relationships [38,39].

In addition, the design of this study will be organized within the Exploration, Preparation, Implementation, and Sustainment (EPIS) framework [40]. The EPIS framework specifies internal and external factors that contextualize the adoption and delivery of services in complex systems such as research trials. Phases 1 and 2 of the study will focus on the exploration and preparation stages by creating adapted intervention packages and evaluating an optimal intervention package. Phase 3 will focus on implementation and sustainment phases of the model, and will primarily be informed by outcome analyses, cost analyses, and feedback from study participants.

The interventions to be adapted are video-based CT, delivered in a dyadic format, along with an individual-level single session of motivational interviewing focused on assisting with identification and development (MI-AID) of sexual goals and communication skills. Video-based communication skills training has received broad support in the literature [41]. The use of videos to reinforce skills related to the negotiation of sexual safety is supported by meta-analytic findings that video content enhances the effectiveness of HIV-prevention interventions that target behavioral skills [42]. This approach builds upon established cognitive-behavioral approaches to assertive communication skill building [43]. Consistent with the results of studies examining the effectiveness of behavioral models, our videos will depict models of positive (exemplary) and negative (nonexemplary) communication [41,44] exchanges between YMSM couples in age-relevant contexts discussing issues related to sexual health and HIV prevention.

Motivational interviewing (MI) has empirical support for its success in reducing adolescent risky sex and sexually transmitted infections (STIs) [32,45-47], including earlier iterations of MI for adolescent HIV risk reduction that are now recognized as empirically supported programs by the Office of Adolescent Health [48]. In line with this team’s prior work in this area [30,32], this intervention has been updated to enhance communication development, particularly for young adolescents who are new to the relationship context. Thus, new developments to this intervention specifically target enhancing nascent communication around HIV risk reduction within young dyads. A single-session MI intervention is used here because it has been shown to have the best possible reach with high-risk youth [45,47]. Not only does prior work underscore that individuals in this age group enjoy and are responsive to MI [46], meta-analyses continue to support the effectiveness of the approach for reducing youth health risk behaviors [49-51].

The MI-AID session begins with an initial introduction describing the session itself and its relationship to the upcoming dyadic HIV testing session with their sexual partner (CHTC). The youth is told that this is an opportunity to identify individual preferences and goals related to sexual agreements, biomedical prevention (pre-exposure prophylaxis, PrEP or post-exposure prophylaxis, PEP), thoughts and preferences on HIV/STI testing, and condom use before discussing these with their partner. The provider’s objective is to both elicit, potentially for the first time, for some youth, the adolescent’s goals and preferences in these domains, as well as to learn how to successfully express those goals and preferences in a relationship context. Active, iterative practice is utilized to ensure that the youth feels fully prepared to transition into the dyadic components of the intervention, including the CT and CHTC sessions.

### Overview and Aims

The purpose of this comparative effectiveness trial (CET) was to test the additive benefit of adjunct treatment components tailored for YMSM, which enhance communication skills before completion of CHTC. The intervention tests a continuum of prevention packages including CT videos with MI-AID before entering into the dyadic intervention components, including CHTC.

## Methods

### Overview of Content and Delivery

This study (We Test, Adolescent Medicine Trials Network 156) is part of the Scale It Up program as described in the overview paper in this issue [52] and will take place in 3 phases. In phase 1, tailored CT videos and MI-AID modules will be developed for partnered YMSM aged 15 to 19 years using information gathered from qualitative interviews with YMSM and SRV site staff. In phase 2, a pilot randomized controlled trial (RCT) will compare a continuum of CHTC packages: CHTC alone; CT videos viewed by the couple in addition to CHTC; and an individually administered MI-AID, along with CT videos and CHTC. Phase 3 will test a sustainable model of CHTC implementation in real-world adolescent HIV clinics. The phase 3 RCT will compare CHTC as usual to the intervention package found most effective in phase 2.

### Eligibility

YMSM who express interest in We Test are assessed for eligibility by completing a brief screener. In phase 1, eligible participants must be (1) cis-male gender identity; (2) aged 15 to 19 years, (3) in a relationship, dating, or seeing a cis-gender male (regardless of relationship duration) with whom they have or anticipate having sex, (4) HIV-negative or status unknown, and (5) able to complete a qualitative interview by Skype, Facetime, or phone. YMSM aged 15 to 17 years must indicate that their partner is aged 15 years or older, and the age difference between partners cannot exceed 2 years. YMSM aged 18 or 19 years may participate if their partner is less than 2 years younger or older than 18 years. Potential participants will be excluded if they are unable to communicate in English; their mental, physical, or emotional capacity does not permit them to complete the protocol as written; they display current suicidal or homicidal ideation; or they are not exerting autonomy over participation (eg, they report that someone forced them to participate in the study).

In phases 2 and 3, study inclusion criteria include (1) at least one partner must be aged 15 to 19 years, (2) both partners identify as cis-male; (3) at least one partner must be HIV-negative or status unknown, (4) partners must be sexually active together or indicate that they plan to have sex together in the future, and (5) both relationship partners must agree to attend an assessment together at an SRV. Similar to phase 1, if either YMSM is aged 15 to 17 years, then the age difference between partners cannot exceed 2 years. YMSM who are aged 18 or 19 years may participate in the study with a partner who is less than 2 years younger or one who is older than 18 years. Potential participants will be excluded if either partner is unable to communicate in English; their mental, physical, or emotional capacity does not permit them to complete the protocol as written; they display current suicidal or homicidal ideation; or they report that someone forced them to participate in the study. Participants from phase 1 may be eligible for participation in later phases.

### Recruitment and Screening

Leveraging existing Scale It Up infrastructure, participants will be recruited and screened for eligibility via 4 different SRVs in phase 1, 2 SRVs in phase 2, and 4 SRVs in phase 3 that provide HIV testing and prevention services to YMSM (Table 1). All SRVs have extensive relationships with the gay, lesbian, bisexual, and transgender communities; community service organizations; health service organizations; and providers for MSM. This may be supplemented by Web-based advertising conducted through Scale It Up’s Recruitment and Enrollment Center (REC). Although advertising will be distributed through the REC, they will be targeted to the geographic areas surrounding the SRVs for this project and will contain information about participation at the SRV.

In phase 1, partnered YMSM will complete a brief Web-based screener. Eligible participants will subsequently be contacted via email or telephone to schedule a qualitative interview. YMSM will receive written consent information before completion of the screener. Those participants who screen eligible and schedule an interview will receive verbal consent information. Similarly, SRV staff will receive written information about the study as part of recruitment materials and will be provided verbal consent information before completion of their interview. A waiver of consent or assent will be obtained to reduce barriers to participation and prevent the need to capture a physical signature in a study conducted remotely. In phases 2 and 3, couples will be screened through an index-case approach. In this approach, 1 partner in the couple will be asked to provide screening information about himself and his partner. If the couple is preliminarily eligible based upon the report of the index partner being screened, that index partner will be asked to schedule a baseline appointment at a time both he and his partner can attend. A waiver of parental consent will be obtained for this study. At the baseline appointments, YMSM partners will be consented privately in separate rooms. A research assistant will review written consent information and obtain consent or assent from both partners before completion of the baseline assessment. Contact information for the recruited (nonindex) partner will be collected at this point and added in REDCap for tracking purposes.

In addition, the Center for HIV Educational Studies and Training (CHEST) will assist in referring potentially eligible participants to the We Test study through existing online recruitment efforts. CHEST utilizes the Hunter College Institutional Review Board (IRB)–approved online master screener (OMS) to preliminarily screen individuals who are interested in participating in studies being conducted through CHEST and live in 1 of the target cities. If an individual is preliminarily eligible for a study, the individual is asked to provide contact information to CHEST for follow-up. For phase 1, the OMS will be used to link potentially eligible participants to the study-specific screener; however, most participants will be recruited through ads that link potential participants directly to the study-specific screener, bypassing the OMS.

For phases 2 and 3, the OMS will only be used to refer potentially eligible YMSM to HIV testing sites by sending them an email referral informing them about the We Test study and where to go to determine eligibility after completing the OMS. The contact information collected through the OMS will not be provided to the SRVs. However, We Test SRVs will be made aware that a potentially eligible YMSM has been referred to their testing services for the study. The OMS, in this study, will primarily be used as a referral mechanism, directing participants to which study they may be eligible for, including We Test. YMSM who complete the OMS and screen preliminarily eligible and who then are referred and subsequently make contact with their SRV will take the We Test–specific screener via the SRV to further establish eligibility. It will be the duty of the SRV site staff to coordinate with the YMSM couples to schedule the baseline appointment.

**Table 1:** Scale It Up subject recruitment venues.

Sites		Location
**Phase 1**		
	CHEST^a^	New York, NY
	Wayne State University	Detroit, MI
	University of Miami	Miami, FL
	San Diego Lesbian, Gay, Bisexual, and Transgender Community Center	San Diego, CA
**Phase 2**		
	CHEST	New York, NY
	Wayne State University	Detroit, MI
**Phase 3**		
	CHEST	New York, NY
	Wayne State University	Detroit, MI
	University of Miami	Miami, FL
	San Diego LGBT Community Center	San Diego, CA

^a^CHEST: Center for HIV Educational Studies and Training.

In phases 2 and 3, community health workers (CHWs) who conduct standard of care HIV testing services for the We Test SRVs will be trained to provide information about the We Test study after HIV-negative results are delivered at their site or in the field at mobile testing events. The CHW will be trained in appropriate and ethical methods of recruiting participants in clinical settings and in the field. To minimize the risk for coercion, the staff member and the study information will emphasize the optional nature of participation and that it will not affect, in any way, their access to health care services. If the potential participant is interested in finding out if he is eligible for We Test after learning about the study, the CHW will provide an iPad with a secure Web-based REDCap We Test Study Screener (IRB approved). The confidential We Test Study Screener will be completed on the iPad by the potential participant, and no one will be able to see the responses; the iPad will only indicate whether the potential participant is eligible.

YMSM who screen ineligible for the study do not need to provide contact information and may screen again after 30 or more days provided they tested HIV-negative at the site in the past 90 days or they again test HIV-negative at the site. Participants are also able to rescreen should they test HIV-negative at the site or through mobile testing efforts associated with the site.

### Study Design

#### Phase 1

**Figure 1:**
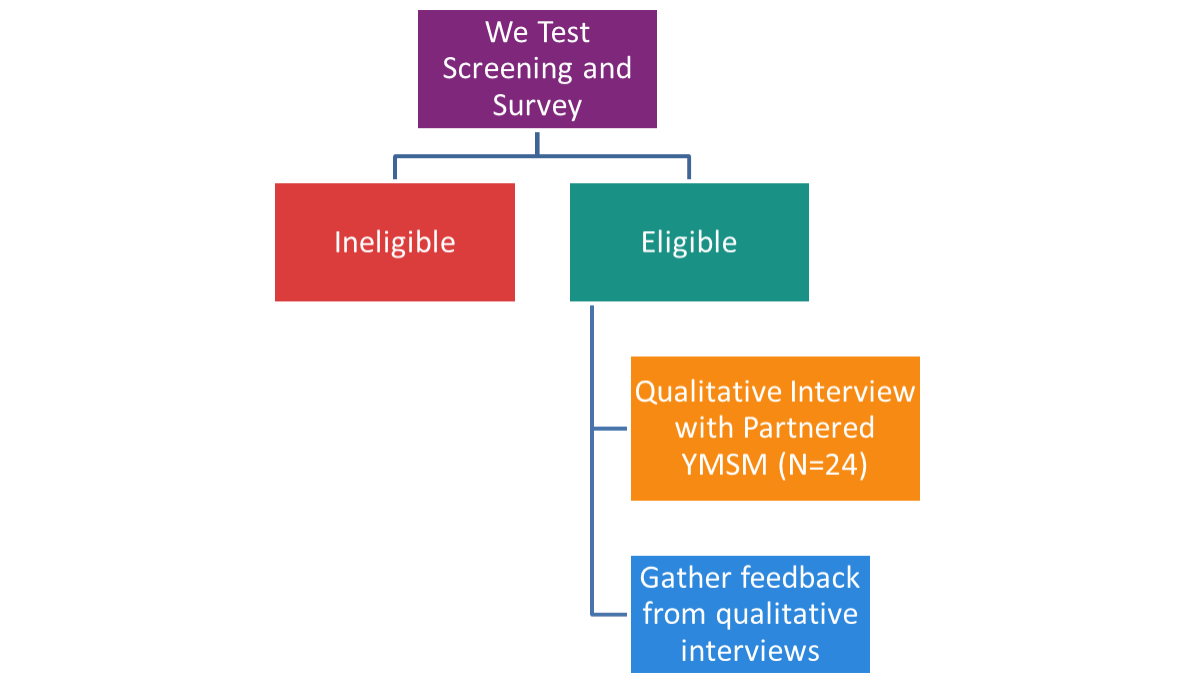
Phase 1 study design. YMSM: young men who have sex with men.

Phase 1 aims to develop tailored CT videos and MI-AID modules for partnered YMSM aged 15 to 19 years (see Figure 1). Qualitative interviews with 20 staff from SRVs and 24 partnered YMSM will be conducted to identify barriers and facilitative factors related to receipt of CHTC for youth as well as to examine sexual communication between primary relationship partners and its link to HIV/AIDS in this age group. Interviews with YMSM will focus on assessing youth’s comfort and capacity to identify sexual goals and communicate about HIV testing and prevention. Interviews will be conducted remotely from CHEST via Skype, Facetime, or phone, based on participants’ preference. Both interfaces have been approved by the IRB. Interviews will be audio-recorded using an external recording device. Recordings will be stored on a secure server and subsequently transcribed. Transcripts will be identified only by participant identification and all proper names will be removed. Participants will receive US $50 each as a compensation for completing this component (see Table 2).

Data gathered from these staff and partnered YMSM qualitative interviews will inform the development of the CT videos and MI-AID. A codebook will be used to structurally and thematically code qualitative output from these interviews using established qualitative methodology. A total of 2 analysts will independently code a subset of the transcripts until intercoder agreement of >90% is reached. All transcripts will then be coded by one of the 2 analysts. Afterward, iterative thematic analyses will be conducted to identify major trends and themes from the interviews. Using this information, CT videos will be created and the individual-level MI-AID intervention will be adapted.

**Table 2:** Compensation. All compensation is delivered either through Visa or Amazon Gift Card or cash depending upon subject recruitment venue restrictions.

Phase and appointment		Study component	Compensation amount (US $)
**1**		
	Qualitative phone interviews	SRV^a^ staff focus group	20
		YMSM^b^ screener and consent	50
		YMSM qualitative interview	—^c^
**2 and 3**		
	Baseline	Baseline CASI^d^	30
		STI^e^ testing	20
		Intervention session (including HIV testing)	20
	1-month follow-up	CASI	50
	3-month follow-up	CASI	30
		HIV testing	10
		STI testing	10
	6-month follow-up^f^	CASI	50
	9-month follow-up^f^	CASI	30
		HIV testing	10
		STI testing	10

^a^SRV: subject recruitment venue.

^b^YMSM: young men who have sex with men.

^c^Not applicable.

^d^CASI: computer-assisted self-interview.

^e^STI: sexually transmitted infection.

^f^Indicates phase 3 only.

#### Phase 2

**Figure 2:**
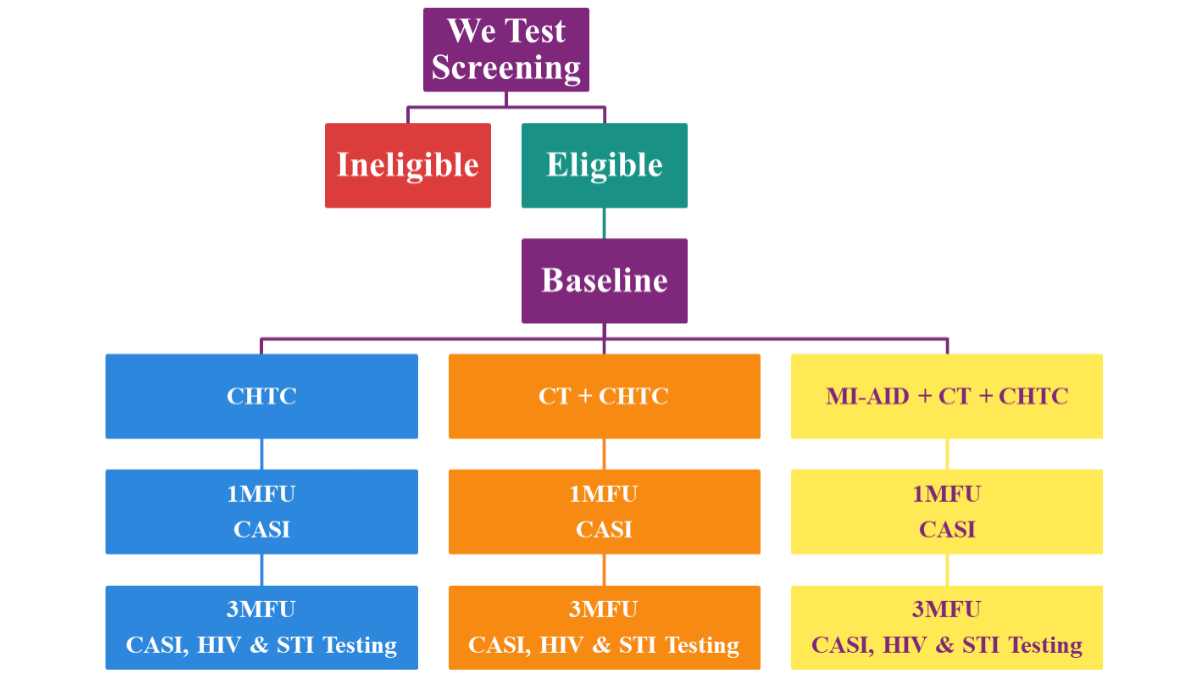
Phase 2 study design. CASI: computer-assisted self-interview, CHTC: couples HIV testing and counseling; CT: communication training; MFU: monthly follow-up; MI-AID: Motivational Interviewing focused on Assisting with Identification and Development; STI: sexually transmitted disease.

In this phase, a pilot RCT will be conducted to evaluate the acceptability and feasibility of adjunct intervention components. This initial trial will recruit 36 couples who will be randomized to 1 of 3 conditions: (1) CHTC only; (2) couples’ joint observation of CT videos and CHTC; (3) each individual within a couple receiving MI-AID, in addition to joint observation of CT videos and CHTC. Youth will then complete follow-up assessments at 1 and 3 months postintervention (see Figure 2).

The main measures that will be tracked are the 4 behavioral indicators of HIV transmission risk behavior (TRB). Two of these are at the individual level: the number of CAS acts with a casual partner in the absence of PrEP and any positive chlamydia or gonorrhea diagnoses in the absence of PrEP. The other 2 behavior indicators of TRB are at the couple level: any sex in the absence of PrEP with a primary partner who reports CAS with a casual partner and any sex in the absence of PrEP with a primary partner who receives a positive chlamydia or gonorrhea diagnosis.

This phase will be completed in 4 steps at 3 time-points—baseline and intervention delivery, followed by 1- and 3- month follow-ups (see Table 3). At the baseline assessment, both members of the couple will attend their local SRV in tandem. Each partner will have STI biomarker specimens collected separately and will independently complete a baseline computer-assisted self-interview (CASI). Each participant will receive US $70 (payable as cash, Visa Gift Card, or Amazon Gift Card based upon SRV restrictions) for completing all components of the baseline assessment (see Table 2). Upon completion of the baseline assessment, couples will be randomized to 1 of 3 study conditions via stratified randomization procedure using Qualtrics. Youth will be stratified by city, age (whether or not both members of the couple are younger than 18 years), and racial composition (if 1 member of the couple identifies as a racial or ethnic minority).

After randomization, the interventions will be delivered by CHWs, former providers of individual HIV counseling and testing services, health educators, and/or trained program peers. The CHTC protocol [53,54] consists of a 25- to 40-min session made up of 8 steps that couples will complete together: (1) description of CHTC, (2) description of HIV testing procedures, (3) exploration of the couples’ relationship, (4) assessment of the couples’ reasons for testing and HIV risk concerns, (5) clarification of sexual agreements, (6) reporting HIV test results, (7) prevention planning or linkage to HIV care, and (8) referral.

The second study condition adds on the CT video intervention. In this intervention, couples will view the 20-min CT video together. This is to provide an added layer of communication training around sexual agreements and HIV testing. The partners will then work together on a brief, 5-min survey about the video content to evaluate treatment receipt.

The third condition adds individually received MI-AID, in addition to the jointly viewed CT video and joint CHTC. In MI-AID, each partner will meet one on one with the CHW for 30 min to address the development of sexual communication. Using the MI-based protocol, the CHW will assist the youth in identification of their own goals for sexual agreement, HIV testing, STI testing, and PrEP/PEP use and assertive communication. In addition, they will use iterative role playing practice in communicating target goals.

**Table 3:** Schedule of assessments, phases 2 and 3.

Component	Baseline	1-month follow-up	3-month follow-up	6-month follow-up^a^	9-month follow-up^a^
**Informed consent**	X^b^	—^c^	—	—	—
**CASI^d^**	X	X	X	X	X
**HIV testing**	X	—	X	—	X
**STI^e^ testing**	X	—	X	—	X
**Randomization**	X	—	—	—	—

^a^Phase 3 only.

^b^X: relevant protocol components at each assessment point.

^c^Not applicable.

^d^CASI: computer-assisted self-interview.

^e^STI: sexually transmitted infection.

Follow-ups will occur at 1 and 3 months post-intervention in phase 2. In the follow-up assessments, participants will complete a Qualtrics CASI, with the 3-month follow-up including rapid HIV testing and STI testing. These follow-ups will be completed individually to facilitate the retention of all participants even if the relationships dissolve. Participants will receive US $50 at both the 1- and 3-month follow-up upon completion of all follow-up components (payable as cash, Visa Gift Card, or Amazon Gift Card based upon SRV restrictions). Those participants who test positive for STIs will be referred for treatment following the standard procedures of the SRV.

#### Phase 3

Phase 3 will build upon the results of the pilot RCT conducted in phase 2. Phase 3 will also gather feedback from staff at our SRVs. EPIS qualitative interviews will be conducted with staff (n=20) from 4 SRVs, namely CHWs, providers, supervisors, and administrators who have experience with the partnered YMSM and CHTC. The objective of these interviews is to elicit staff perspectives on (1) the nature of content included in adapted CT and MI-AID interventions, (2) structural considerations that must be accommodated in the intervention protocol, and (3) developmental concerns of delivering CHTC to youth younger than 18 years. The focus will be on identifying factors that can ultimately enhance intervention acceptability and sustainability, while retaining core elements of all intervention components. The EPIS protocol paper in this issue [55] outlines how these components are captured for analysis. SRV staff will receive US $20 as compensation in the form of an Amazon Gift Card for their time for completing the EPIS interview and survey (see Table 2).

**Figure 3:**
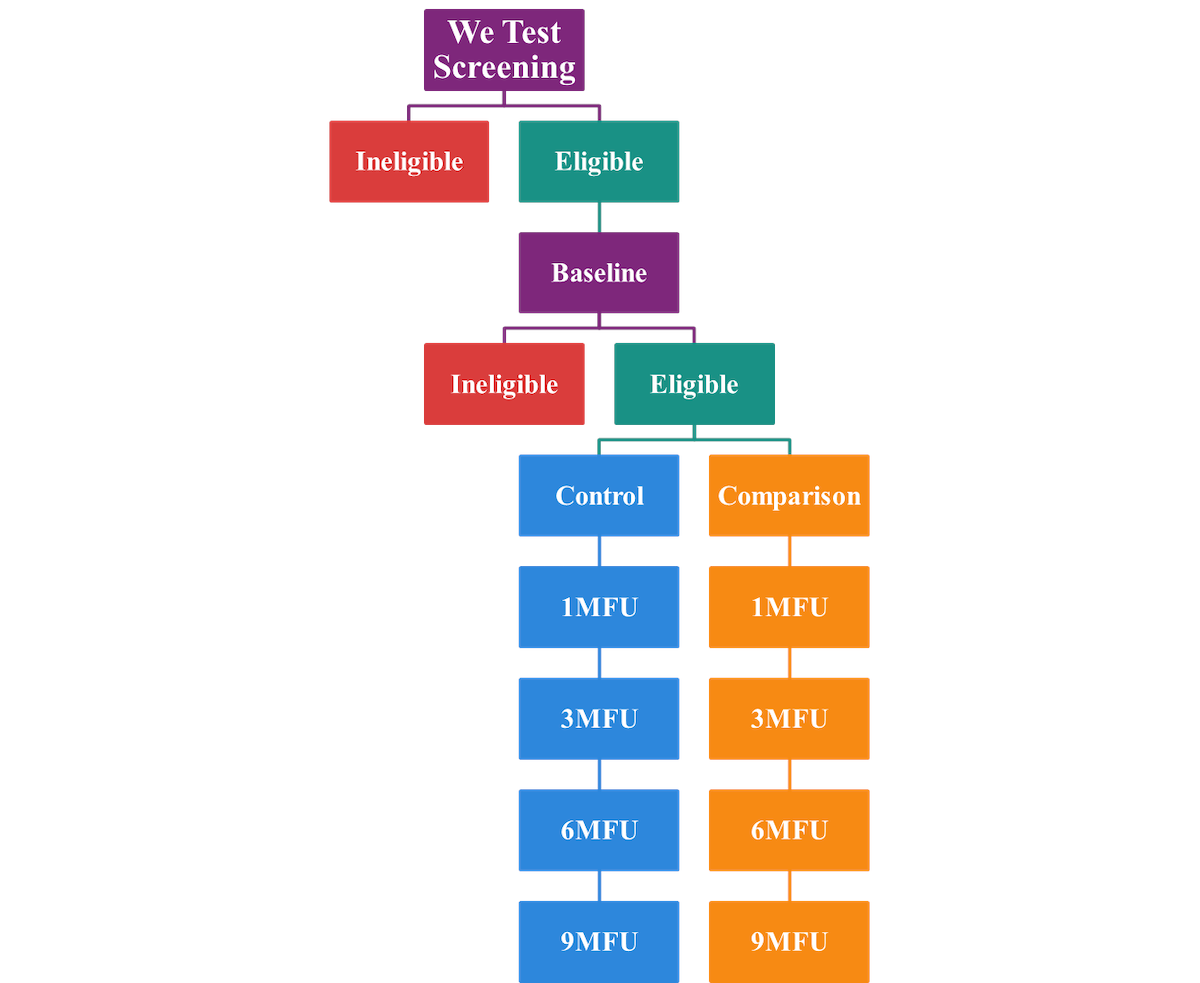
Phase 3 study design. MFU: monthly follow-up.

Phase 3 will enroll 144 couples from 4 SRVs into the full RCT (see Figure 3). In phase 3, couples will be randomized to 1 of only 2 conditions. Although the control condition will be CHTC delivered as usual, the comparison condition will include the optimal intervention package indicated during our phase 2 adaptation. This condition may therefore involve viewing CT videos only before CHTC, individual MI-AID before CHTC only, or both depending upon results and feedback obtained in phase 2. Youth in this RCT will complete follow-up assessments at 1, 3, 6, and 9 months (see Table 3).

Eligibility criteria during phase 3 are the same as phase 2. The same recruitment, consent, and baseline assessment procedures will be utilized in both phases. Although there are additional 6- and 9-month follow-up assessments in phase 3, the procedures remain the same.

### Training of Interventionists

Following phase 1, to prepare for the rollout of the CET, there will be a week-long in-depth training on all study procedures. This training will include members of the 2 SRVs who will participate in phase 2. Any staff who cannot attend because of nonresolvable travel restrictions will participate via Skype. This training will include 2-day training in CHTC, 2-day training on MI-AID, and 1 day dedicated to reviewing and practicing protocol delivery, including administration of the CT videos. Throughout training and fidelity-monitoring activities, the implementation team will work to document CHW and supervisor performance and feedback on intervention materials and delivery (reflecting the exploration and preparation phases of EPIS). At the conclusion of the CET, the project implementation team will work closely with the implementation science core to package training and intervention materials in a manner that supports the dissemination of CHTC and adjunct components shown to be effective.

We will use a train-the-trainer model, where centralized training will be conducted for CHWs and their supervisors on the implementation and delivery of the intervention materials, with the goal that supervisors will serve as local implementation *champions* by instructing and overseeing implementation within their SRV. To maximize accuracy, consistency, and fidelity of intervention delivery, each intervention (CHTC, CT, and MI-AID) will be codified into manuals. This training sequence will be offered again before phase 3 to bring the 2 new SRVs into the study and update staff at existing SRVs.

### Fidelity Monitoring

All sessions will be audio-recorded for the purposes of systematic supervision so that fidelity can be assessed and interventionist drift prevented. A team of Motivational Interviewing Treatment Integrity (MITI) [56] coders trained in the assessment of MI, along with research assistants trained in the assessment of CHTC fidelity, will ensure the fidelity of intervention packages. These MITI coders will review the first 10 sessions completed by each CHW and a random selection of sessions for the remainder of the trial (approximately 1 in 4 of sessions completed by each CHW over the entire trial). Coding of all initial sessions will ensure fidelity across interventionists at the start of the trial, and subsequent coding will identify any interventionist drift that occurs for the remainder of the trial. Tapes will be coded to ensure the presence of essential elements of the intervention. When interventionists exhibit low levels of intervention integrity or significant drift, the feedback will be relayed to their on-site supervisors. The training team will then work with the local supervisor to develop a remediation plan to bring the CHW back up to parity with other interventionists.

### Reporting Adverse Events

In addition, the site protocol lead (PL) is responsible for the detection and documentation of events meeting the criteria and definition of an adverse event (AE) or serious adverse event (SAE). Data for monitoring participants’ safety will be captured within the REDCap database as part of the required study data. Site study staff may ask questions concerning AEs via the Scale It Up query system but must formally report them via email and REDCap. Information on unexpected events including SAE will be reported as per the policy of Scale It Up’s single IRB.

Information to be collected includes the nature, date of onset, stop date, intensity, duration, treatment, causality, and outcome of the event. Site PLs should follow usual clinical practices at their institutions for reporting serious, unexpected events related to standard of care. SAEs that occur after 30 days after completion of the study will be collected only if they are considered by the PL to be related to study participation. In addition, any AE resulting in potential participant withdrawal must been reported to the Scale It Up REC before participant withdrawal when possible.

## Results

Participant recruitment for phase 1 qualitative interviews with partnered YMSM occurred between December 2017 and October 2018. The target start date for the phase 2 enrollment is November 2018, phase 3 to begin in September 2018, and all 3 phases will finish by 2021.

### Quantitative Analysis Plan

The primary hypothesis is that because of developing skills in self-management and assertive communication, inclusion of adjunct components will be associated with clinically significant decreases in HIV TRB as compared with partnered YMSM who receive CHTC (only). Secondarily, we propose that these intervention effects will be mediated by assertive communication skills. As stated above, we focus on 4 behavioral indicators of TRB. At the individual level, we examine **(**1) number of CAS acts with a casual partner in the absence of PrEP and (2) any positive chlamydia or gonorrhea diagnoses in the absence of PrEP. At the couple level, we will examine (1) any sex in the absence of PrEP with a primary partner who reports CAS with a casual partner and (2) any sex in the absence of PrEP with a primary partner who receives a positive chlamydia or gonorrhea diagnosis. Any missing data and additional covariates will be informed by attrition analyses before primary analyses.

### Analytic Plan

All primary outcome variables will be tested in the context of a multilevel growth model, which accounts for the nesting of individuals within couples. To capture within-individual change over time, we will utilize a latent growth curve approach to modeling follow-up data. At the individual level (level I), models will include an intercept and linear slope component to represent the initial value and change over time in each participant’s outcome. We will explore the inclusion of quadratic components as indicated by model fit. Mplus provides the flexibility to accommodate count and dichotomous outcomes. Growth factors will then be regressed on intervention condition at the couple level (level II), and the effect of the intervention will be evaluated by examining the regression coefficient (and associated *P* value) associated with intervention condition for each of these factors.

Secondary analyses of individually reported self-management and dyadic functioning as potential mediators of the intervention’s effect on and TRB will specify growth factors for self-management, dyadic functioning, and communication skill scores during the follow-up period [57]. In this manner, growth factors for the outcome can be regressed on growth factors for the putative mediator. Intervention effects (a couple-level predictor) will be determined by examining regression coefficients associated with intervention in the prediction of growth factors for both the outcome of interest and mediator. For significant direct effects, indirect pathways from intervention communication will be tested using bootstrapping tests of mediation. Where outcome distributions prevent bootstrapping, we will utilize a model constraint approach to evaluate the significance of indirect effects. The product of constituent direct effects is constrained as zero. The overall model fit under this constraint is compared with one where the constraint is not specified. A statistically significant reduction in fit associated with constraint represents evidence that indirect effects differ from zero [58].

### Power Analysis

Consistent with the intervention development goals of phase 2, we are not powered to detect significant between-condition differences in primary outcomes for that phase. Power analyses for phase 3 were conducted based on our preliminary pilot data extracted from a similar study (R34 DA036419; PI Starks) testing adjunct CHTC components in emerging adult gay male couples aged 18 to 29 years. Preliminary results from the 3-month wave of data collection (the most distal available with sufficient data to estimate effects at the time of this submission) suggested that viewing CT videos before CHTC was associated with a 56% decrease in the odds of CAS with a casual partner (relative to CHTC alone) among HIV-negative participants not on PrEP. Of particular relevance to our mediation hypotheses, viewing CT videos before CHTC was associated with a 5- to 6-point decrease in avoidant communication as measured by the Communication Patterns Questionnaire (CPQ). In turn, CPQ avoidant communication scores had a significant positive association with CAS with casual partners among HIV-negative men not on PrEP (*expB*=1.06, *P*<.01). Separately, our previous study of brief MI interventions with YMSM suggests that the receipt of MI is associated with as much as an 83% within-condition reduction in CAS over time and a 24% reduction in the odds of CAS with a casual partner compared with an attention-matched psychoeducation control condition [59].

These preliminary effect sizes were utilized as parameters in power analyses using a Monte Carlo simulation approach in Mplus (version 7.3) [58]. This approach provides a direct estimation of power while modelling both the multilevel structure of the data (individuals are nested within couples) and the longitudinal design of the study (each individual provides data at 3 follow-up points). The program generates random samples from the specified population and, within each sample, examines the significance of freed parameters. Power is defined as the proportion of simulated samples in which the freed parameter has a *P* value of less than .05. All models specified a random seed and used 10,000 sample replications. Power analyses conducted based on these preliminary effect sizes suggest that 144 couples (N=288 individuals), anticipating a minimum of 232 individuals retained at 9 months, is adequate to achieve power >.80 for all hypothesized direct effects as well as indirect pathways.

### Equivalency Tests

We will follow standard procedures in cleaning data and examining initial distributional properties (means, SDs, medians, skew, and kurtosis) in addition to graphical summaries (boxplots and density plots). Subsequently, we will evaluate the suggestions of randomization by testing between-condition differences with respect to demographic covariates and primary outcomes reported at baseline. Note, because participants are nested within dyads, these analyses will utilize the generalized estimating equations (GEEs) module within SPSS to control for the nonindependence of observations and specify outcome distributions that are appropriately matched to the variables of interest.

Finally, we will conduct an analysis of attrition to determine if dropout at each follow-up timepoint is associated with (1) demographic variables assessed at baseline and/or (2) drug use or TRB outcomes assessed at baseline. At each wave, we will utilize GEE models to evaluate whether those participants retained at the given wave differ significantly with respect to demographic or baseline outcome values compared with those who were not retained. As with the analyses of randomization success, the use of GEE permits analyses to control for the nesting of participants within couples and specify outcome distributions that are matched to variables of interest. Factors that are observed to covary significantly with attrition will be incorporated as covariates in outcome analyses. Mplus has a variety of options for handling partial attrition including full-information maximum likelihood estimation [60]. Nonrandom and consequential missingness can also be modeled directly through the addition of latent variables, which account for the probability of missingness at any timepoint. Where indicated, we will explore the use of these procedures in the analyses described below.

## Discussion

### Summary of Key Innovations

The primary innovation of this multilevel CET lies in the novel packaging of existing interventions in a manner that addresses the specific developmental needs of YMSM. Although MI-AID, video-based CT, and CHTC have individually established efficacy, this study will be the first to evaluate a continuum of prevention packages, which combine these components.

In addition, this multilevel intervention seeks to leverage the power of the dyadic processes to enhance motivation for HIV testing and prevention (including biomedical prevention). Our adjunctive components (CT and MI-AID) are specifically intended to enhance self-management skills (eg, assertive communication), which are essential to effectively engage relationship partners in collaborative sexual goal development and problem solving. The underlying assumption of this strategy is that improvements in dyadic functioning will lead to reductions in sexual risk for both individuals within the relationship.

Another strength lies in the inclusion criteria, which were constructed to accommodate the broadest possible range of sexual partnerships at this exciting developmental stage. The study does not impose any requirement on relationship duration. Given the dynamic and potentially experimental nature of relationships at this stage, we have utilized a liberal definition of relationship. YMSM are not required to identify their partner as a boyfriend or indicate *being partnered*. Instead, they may identify themselves as dating, or experimenting with a relationship, and still be eligible as long as the other person involved is a cis-gender male with whom they either are or may be sexually active. This inclusive stance toward relationships is consistent with the CHTC protocol [53,54], created to be applicable for any couple who has or *intends to have* a sexual relationship.

### Limitations and Conclusions

There are a few limitations to this study. First, this study requires that participants are able to communicate in English. As a result, nonfluent English speakers—such as those who have recently immigrated or those who live in predominately non-English speaking communities—are unable to participate. This misses out on key sectors of the YMSM population, particularly as our SRVs located in New York, Detroit, Miami, and San Diego are racially, ethnically, and linguistically diverse areas. In particular, Spanish-speaking YMSM cannot participate in this study.

In addition, this study is limited in its location. The study takes place in 4 major cities. Although these cities are geographically diverse, the study is unable to adapt its interventions for partnered YMSM from other cities. In particular, YMSM from rural areas away from these cities are unable to participate. The effectiveness of these intervention packages may or may not vary depending on a YMSM’s locale.

Ultimately, this study is an innovative design which not only incorporates community feedback to develop interventions but also uses 2 CETs to determine the most effective continuum of interventions for partnered YMSM. This study will be the first to combine MI-AID, CT video, and CHTC. Packaging these trainings with CHTC may enhance prevention for the uniquely vulnerable population of partnered YMSM.

## Abbreviations

AE: adverse event
ATN: Adolescent Medicine Trials Network
CAS: condomless anal sex
CASI: computer-assisted self-interview
CET: comparative effectiveness trial
CHEST: Center for HIV Educational Studies and Training
CHTC: couples HIV testing and counseling
CHW: community health workers
CIT: couples interdependence theory
CPQ: Communication Patterns Questionnaire
CT: assertive communication training
EPIS: Exploration, Preparation, Implementation, and Sustainment
GEE: generalized estimating equation
IRB: Institutional Review Board
MI: motivational interviewing
MI-AID: motivational interviewing focused on assisting with identification and development of sexual goals and communication
MITI: Motivational Interviewing Treatment Integrity
MSM: men who have sex with men
OMS: online master screener
PEP: post-exposure prophylaxis
PL: protocol lead
PrEP: pre-exposure prophylaxis
RCT: randomized controlled trial
REC: Recruitment and Enrollment Center
SAE: serious adverse event
SRV: subject recruitment venue
STI: sexually transmitted infection
TRB: transmission risk behavior
YMSM: young men who have sex with men
